# First Complete Genome Sequence of *Pepper vein yellows virus* from Australia

**DOI:** 10.1128/genomeA.00450-16

**Published:** 2016-05-26

**Authors:** Solomon Maina, Owain R. Edwards, Roger A. C. Jones

**Affiliations:** a School of Plant Biology and Institute of Agriculture, Faculty of Science, The University of Western Australia, Crawley, Western Australia, Australia; b Cooperative Research Centre for Plant Biosecurity, ACT, Canberra, Australia; c CSIRO Entomology, Floreat Park, Western Australia, Australia; d Department of Agriculture and Food Western Australia, South Perth, Western Australia, Australia

## Abstract

We present here the first complete genomic RNA sequence of the polerovirus *Pepper vein yellows virus* (PeVYV) obtained from a pepper plant in Australia. We compare it with complete PeVYV genomes from Japan and China. The Australian genome was more closely related to the Japanese than the Chinese genome.

## GENOME ANNOUNCEMENT

To examine possible connectivity between viruses infecting crops in Australia and Asian countries to Australia’s north, pepper (*Capsicum annuum*) viruses from northwest Australia were studied. During July 2015, leaf samples were collected from pepper plants growing on a farm in the Ord River Irrigation Area of the tropical East Kimberley region. RNA extracts from seven samples were subjected to next-generation sequencing. A complete genome of *Pepper vein yellows virus* (PeVYV) was obtained from sample 12KNX1 from a plant with leaf interveinal yellowing and curling symptoms. PeVYV is a single-stranded RNA virus in the genus *Polerovirus,* family *Luteoviridae*. It occurs in several Southeast Asian countries, including Indonesia, the Philippines, and Thailand, Northeast, South, and West Asia, Africa, Europe, and North America (1–4). Currently, there are only two other complete PeVYV genomes on GenBank, accession no. AB594828 from Japan and accession no. KP326573 from China (2, 3).

Total RNA was extracted from sample 12KNX1 using a ZR plant RNA MiniPrep kit (Zymo Research, Irvine, CA, USA) treated with RNase-free DNase (Invitrogen, Carlsbad, CA, USA) and measured using Qubit (Invitrogen). RNA integrity was confirmed using RNA screen tape (TapeStation 2200; Agilent Technologies, Santa Clara, CA, USA). Libraries were prepared from total RNA extracts using a TruSeq stranded total RNA sample preparation kit, the Ribo-Zero plant kit (catalog no. RS-122-2401; Illumina, San Diego, CA, USA). The final size and concentration of each library were verified using Qubit and D1000 ScreenTape (TapeStation 2200). Sequencing was by HiSeq 2500 using a TruSeq SBS kit version 4 (Illumina) with 151 cycles of paired-end reads. The reads were assembled and genomes annotated using CLC Genomics Workbench 6.5 (CLC bio) and Geneious 8.1.7 (Biomatters) (5).

Leaf sample 12KNX1 yielded 12,422,032 reads with a Q30 of 98.12% and, after trimming, 12,420,466 reads remained. *De novo* assembly generated 14 contigs, 73,829 reads mapping to the contig of interest, giving a coverage of 2,545×. The assembled full-length genome of 12KNX1 was 6,244 nucleotides in length, with the six open reading frames (ORFs) typical of the genus *Polerovirus*; ORF0 and ORF2 overlap with ORF1, and ORF4 is included within ORF3 to ORF5. A comparison of the 12KNX1 genomic sequence with the other two available PeVYV genomic sequences revealed that 12KNX1 had pairwise nucleotide identities of 95.4% and 90.9% to AB594828 and KP326573, respectively. The extent of nucleotide sequence divergence among the three genomes was too great to indicate any connectivity among them, but the Japanese genome (GenBank accession no. AB594828) was closer than the Chinese genome (GenBank accession no. KP326573) to the Australian genome (GenBank accession no. KU999109). To establish whether PeVYV was introduced into northern Australia from nearby Southeast Asian countries or vice versa, a comparison of PeVYV genomic sequences from Australia with ones from neighboring Southeast Asian countries would be required.

### Nucleotide sequence accession number.

The sequence was deposited in GenBank under accession no. KU999109 (12KNX1).
